# MicroRNA-584-3p, a novel tumor suppressor and prognostic marker, reduces the migration and invasion of human glioma cells by targeting hypoxia-induced ROCK1

**DOI:** 10.18632/oncotarget.6735

**Published:** 2015-12-23

**Authors:** Hao Xue, Xing Guo, Xiao Han, Shaofeng Yan, Jinsen Zhang, Shugang Xu, Tong Li, Xiaofan Guo, Ping Zhang, Xiao Gao, Qinglin Liu, Gang Li

**Affiliations:** ^1^ Department of Neurosurgery, Qilu Hospital of Shandong University, Jinan, Shandong Province, P.R. China; ^2^ Brain Science Research Institute, Shandong University, Jinan, Shandong Province, P.R. China; ^3^ Department of Neurosurgery, Dezhou People's Hospital, Dezhou, Shandong Province, P.R. China

**Keywords:** microRNA, glioma, motility, hypoxia, prognosis

## Abstract

Here, we report that microRNA-584-3p (miR-584-3p) is up-regulated in hypoxic glioma cells and in high-grade human glioma tumors (WHO grades III–IV) relative to normoxic cells and to low-grade tumors (WHO grades I–II), respectively. The postoperative survival time was significantly prolonged in the high-grade glioma patients with high miR-584-3p expression compared with those with low miR-584-3p expression. miR-584-3p may function as a potent tumor suppressor and as a prognostic biomarker for malignant glioma. However, the molecular mechanisms underlying these properties remain poorly understood. Our mechanistic studies revealed that miR-584-3p suppressed the migration and invasion of glioma cells by disrupting hypoxia-induced stress fiber formation. Specifically, we have found that ROCK1 is a direct and functionally relevant target of miR-584-3p in glioma cells. Our results have demonstrated a tumor suppressive function of miR-584-3p in glioma, in which it inhibits the migration and invasion of tumor cells by antagonizing hypoxia-induced, ROCK1-dependent stress fiber formation. Our findings have potential implications for glioma gene therapy and suggest that miR-584-3p could represent a prognostic indicator for glioma.

## INTRODUCTION

Glioma, which is the most malignant tumor type, accounts for more than 70% of all brain tumors [1]. The most common subtype of glioma is glioblastoma (GBM), with an age-adjusted incidence rate ranging from 0.59 to 3.69 per 100,000 persons [2]. The biological characteristics of GBM primarily include high mortality and recurrence rates [3], uncontrollable invasiveness [4], strong angiogenesis [5], and widespread hypoxia [6]. Hypoxia is a common feature in solid tumors due to their overwhelming progression and relatively inadequate blood supply. Tumor hypoxia is an independent prognostic factor for poor survival [6]. Indeed, numerous studies have suggested that hypoxia activates many cellular processes in tumors, such as proliferation, survival [7], angiogenesis [8], migration, and invasion [9]. Although several mechanisms have been proposed to elucidate the hypoxia-induced migration and invasion of tumor cells, an increasing number of studies have evaluated the roles of highly expressed microRNAs (miRNAs) in mediating the effects of hypoxia.

miRNAs are a class of endogenous 20- to 22-nucleotide (nt)-long, single-stranded non-coding RNAs that have been identified as negative regulators of gene expression at the post-transcriptional level [10, 11]. These small molecules are incorporated into the RNA-induced silencing complex and bind to the seed sequence in the 3′-untranslated regions (3′-UTRs) of their target mRNAs to silence gene translation [11]. Mature miRNAs are processed from longer 70-nt pre-miRNAs by RNase III-like enzymes in the cytoplasm. Pre-miRNAs are long duplex hairpins that contain several bulges or mispairings and that are capped by an apical loop of variable size [12, 13]. Previous studies have suggested that the 5′ arm of the pre-miRNA stem-loop sequence will become a unique, mature miRNA, while the 3′ arm will be degraded rapidly [14]. Nevertheless, growing evidence now suggests that miRNAs encoded by the 3′ arm (miR-3p) exist simultaneously with those encoded by the 5′ arm (miR-5p) and that these two groups of miRNAs target completely different sets of mRNAs because of their disparate sequences even if they originate from the same precursor [15]. Therefore, additional research is warranted to determine the important roles of numerous miRNAs in diverse tumor-related cellular processes, such as origination [16, 17], proliferation [18, 19], angiogenesis [20–22], survival [23], and metastasis [24–26]. In gliomas, various tumor-promoting [27–29] and tumor-suppressing miRNAs [23, 30–33] have been identified. For example, miR-584 has been reported to be a tumor-suppressor in glioma [34] and in other tumors, such as renal cell carcinoma [35] and breast cancer [36]. Surprisingly, all of these studies have focused only on miR-584-5p as the unique mature miRNA form of miR-584 pre-miRNA. However, miR-584-3p, a completely different mature miRNA of miR-584 pre-miRNA, also exists, and its exact role in tumors has not been elucidated.

The present study is the first to report that miR-584-3p is a novel tumor suppressor and a valuable prognostic marker of glioma. First, we demonstrated that miR-584-3p expression was much lower in low-grade glioma patients than in high-grade glioma patients. Furthermore, the postoperative survival time was significantly prolonged in the high-grade glioma patients possessing tumors with high miR-584-3p expression compared with those having tumors with low miR-584-3p expression. Further investigation using glioma cell lines and orthotopic implantation of tumors into a nude mouse model revealed that miR-584-3p reduced the migratory and invasive abilities of glioma cells by targeting the hypoxia-induced kinase ROCK1. The finding of the anti-invasive effect of miR-584-3p suggests that it has an unexpected fundamental tumor-suppressive function in glioma, which partially explains the poor prognosis of the glioma patients with low miR-584-3p expression. Prospectively, miR-584-3p acts as a prognostic biomarker of malignant glioma and is particularly important for patients with WHO III–IV gliomas. Our data highlight the importance of miRNAs in tumor development and provide new insights into the molecular mechanisms underlying tumor progression.

## RESULTS

### miRNA array analysis of differentially expressed miRNAs in hypoxic glioma cell lines and validation of the miR-584-3p expression levels in glioma tumors and cell lines

**Figure 1:**
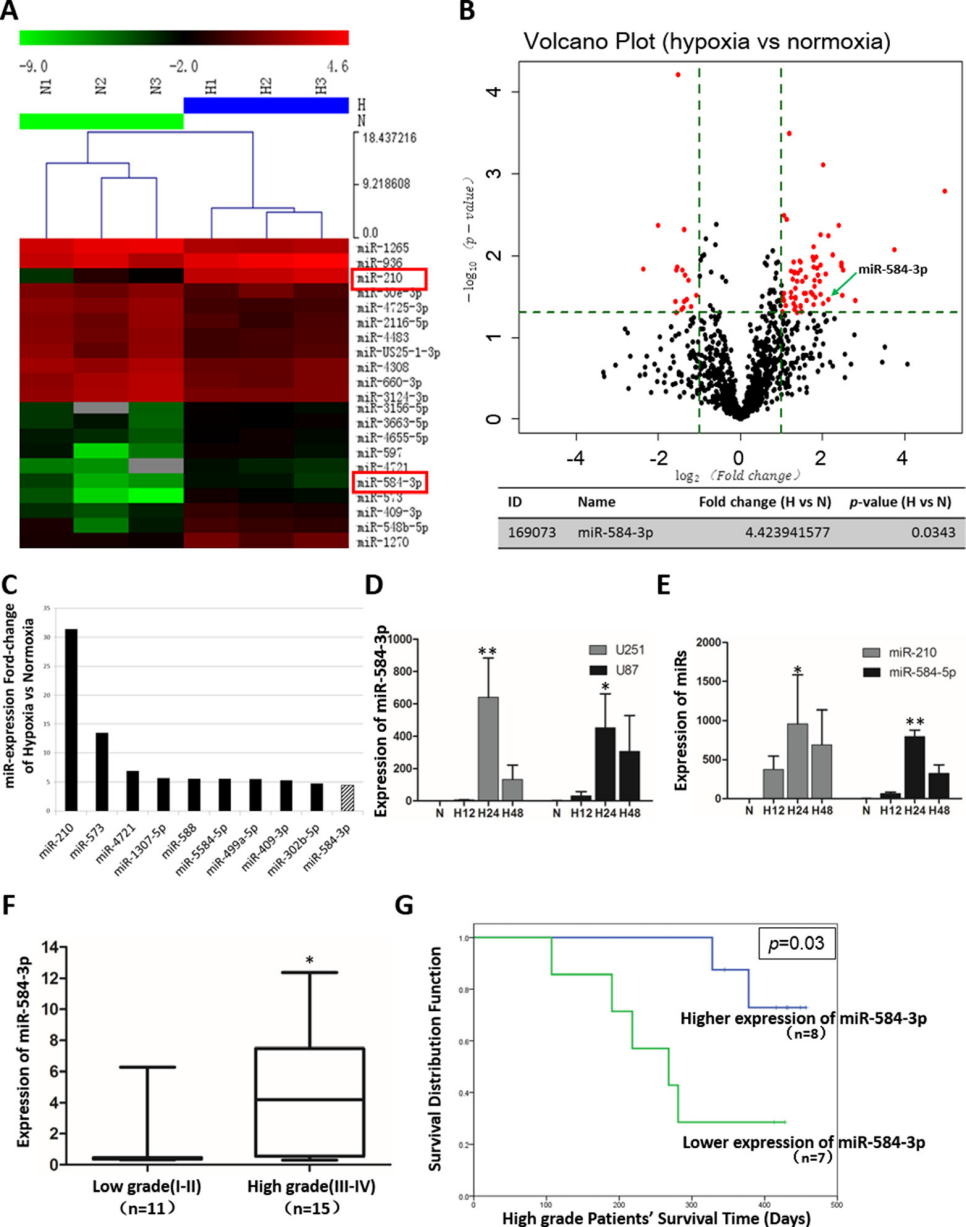
miRNA array analysis of differentially expressed miRNAs in hypoxic glioma cell lines and miR-584-3p expression levels in human glioma tumors and cell lines (**A**) miRCURY^™^ LNA expression array revealed 84 significantly differentially expressed miRNAs (partial data in figure) between normoxic and hypoxic U251 cells. The hypoxic miRNA marker miR-210 and the target miRNA miR-584-3p are indicated. (**B**) Volcano plots were constructed to screen the differentially expressed miRNAs between the two conditions according to the fold changes and *p*-values. The vertical lines correspond to 2.0-fold changes (either up or down), and the horizontal line represents a *p*-value of 0.05. The red dots in the plot represent the significantly differentially expressed genes, including miR-584-3p. (**C**) miR-584-3p was among the top ten modulated genes in hypoxic glioma cells, and it was up-regulated (according to the fold change). (**D**) The expression levels of miR-584-3p in hypoxic U251 and U87 cells (hypoxia treatment for 0, 12, 24, and 48 h) were assessed by quantitative real-time PCR. **p = 0.0084, *p = 0.0131 by Kruskal-Wallis one-way ANOVA. (**E**) The expression levels of miR-210 and miR-584-5p in hypoxic U251 cells (hypoxia treatment for 0, 12, 24, and 48 h) were assessed by quantitative real-time PCR. **p = 0.0035, *p = 0.0267 by Kruskal-Wallis one-way ANOVA. (**F**) The miR-584-3p expression levels in surgically removed glioma tissues from 26 patients (11 patients with WHO grade I–II gliomas and 15 with WHO grade III–IV gliomas) were measured by quantitative real-time PCR. *p = 0.0137 by Mann-Whitney test. (**G**) Prognostic significance of miR-584-3p expression in high-grade glioma patients. The Kaplan-Meier survival curves for the high-grade glioma patients show that low miR-584-3p expression is correlated with poor prognosis. The median miR-584-3p expression level in the 15 high-grade glioma tissue samples was determined by quantitative real-time PCR and selected as the cutoff value, with a log-rank (Mantel-Cox) significance of *p* = 0.03. Abbreviations: miR-584-3p, microRNA-584-3p; H, hypoxia; N, normoxia. The graph shows the mean ± SD and *p < 0.05, **p < 0.01, and ***p < 0.001.

Because hypoxia is an independent prognostic factor for poor survival in glioma patients, we investigated the expression of hypoxia-induced miRNAs by performing high-throughput screening of all genomic miRNAs in normoxic and hypoxic U251 cells using a miRNA microarray, and then we identified several targets with significantly increased expression under hypoxic conditions. This microarray analysis revealed that 84 miRNAs were significantly differentially expressed. The most highly up-regulated miRNA, miR-210, was identified as a novel miRNA marker of hypoxia (Figure 1A). In addition, miR-584-3p was identified as the only one among the top ten up-regulated miRNAs that significantly inhibited glioma cell migration, as we will discuss later (Figure 1B, 1C). To assess significant differences in gene expression between hypoxic and normoxic conditions, we analyzed the data using volcano plot filtering (Figure 1B). We further verified the miR-584-3p expression levels in hypoxic U251 and U87 cells by quantitative real-time PCR and found that the validated expression results were consistent with the microarray results. miR-584-3p expression peaked at approximately 24 h of hypoxia treatment in glioma cells (Figure 1D). The up-regulation of miR-210 indicated the qualified hypoxic conditions, and miR-584-5p expression also increased similarly, which suggested that miR-584-3p was being regulated at the transcriptional level (Figure 1E).

Next, RT-PCR was performed to analyze miR-584-3p expression in clinical samples of surgically removed glioma tissues from 26 patients (Table 1). Interestingly, significant differences in miR-584-3p expression were observed between the low-grade (I–II) and high-grade (III–IV) glioma patients. Consistent with the results obtained for the cell lines, miR-584-3p expression was significantly lower in the low-grade glioma tissues than in the high-grade glioma tissues (Figure 1F), which was possibly because high-grade gliomas possess a more hypoxic microenvironment due to their rapid proliferation. Furthermore, the miR-584-3p expression levels displayed a dispersed distribution among the high-grade glioma patients, who could be divided into higher expression and lower expression subgroups. Next, associated clinical survival information of the patients was analyzed using Kaplan-Meier estimates. Unexpectedly, the subgroup of high-grade (III–IV) glioma patients with high miR-584-3p expression presented a significantly prolonged postoperative survival time (Figure 1G). The above findings raised the intriguing possibility that miR-584-3p could act as a tumor suppressor and could represent a prognostic biomarker of malignant glioma. However, the underlying mechanisms by which miR-584-3p suppresses malignant glioma progression remain unclear.

**Table 1:** Demographic parameters of patients participating in the study

		NO. of Patients	*N*%
Assessable			
	Entered	26	100%
	For survival analysis	15	57.69%
Gender			
	Male	20	76.92%
	Female	6	23.08%
Age (years)			
	Median (range)	45.04 (5∼70)	
Pathological type			
	Astrocytoma	7	26.92%
	Anaplastic astrocytoma	3	11.54%
	Pilocytic astrocytoma	1	3.85%
	Oligodendroglioma	3	11.54%
	Anaplastic oligodendroglioma	2	7.69%
	Glioblastoma	9	34.62%
	Dysembryoplastic neuroepithelial tumor	1	3.85%
WHO tumor grade at diagnosis			
	I	2	7.69%
	II	9	34.62%
	III	6	23.08%
	IV	9	34.62%

### miR-584-3p knockdown enhanced the migratory and invasive capacities of human glioma cells and aggravated the pro-migratory and pro-invasive effects of hypoxia

**Figure 2:**
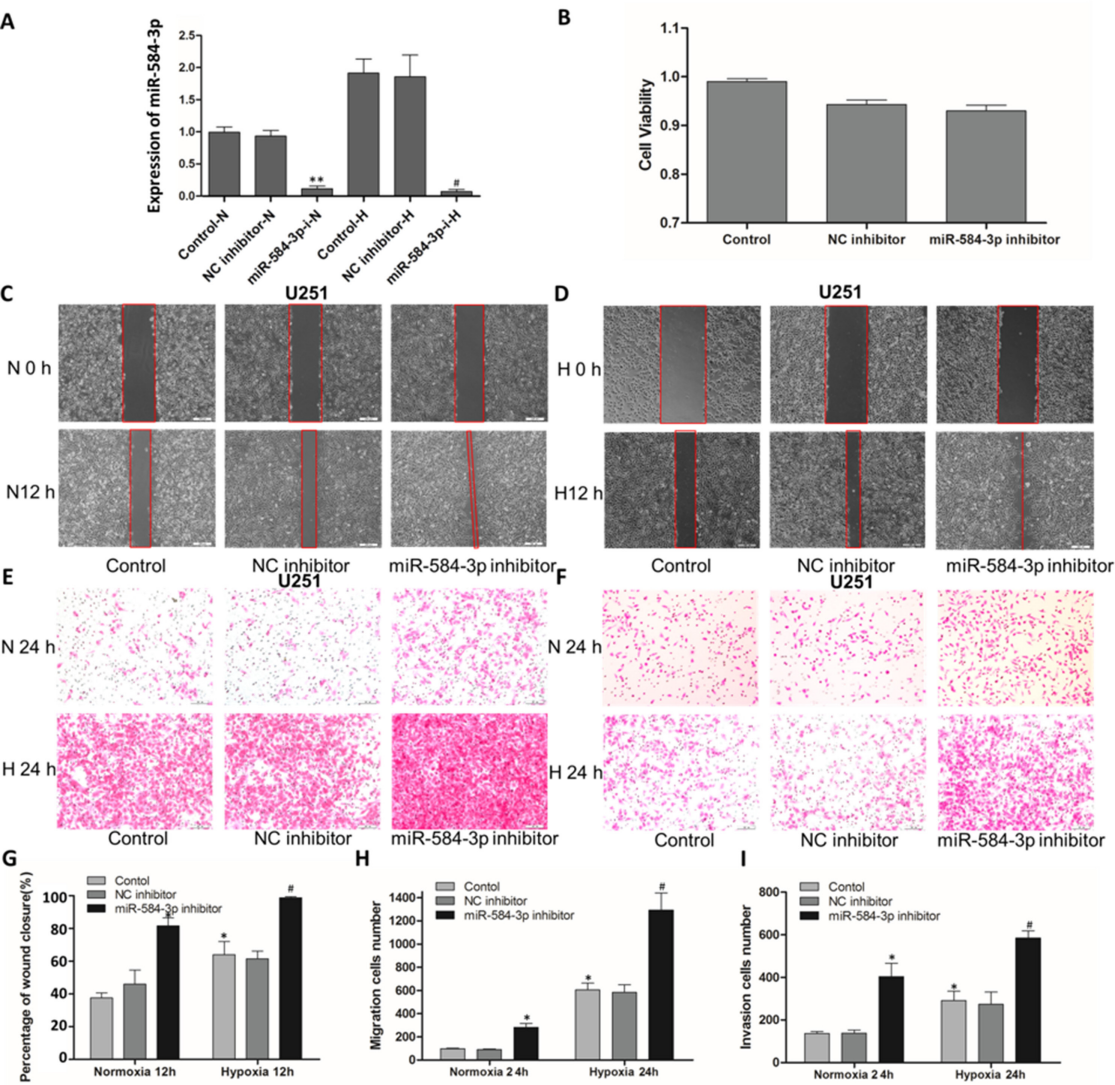
miR-584-3p knockdown enhanced the migratory and invasive capacities of human glioma cells, as shown by wound-healing and Transwell assays (**A**) The knockdown efficiency of the miR-584-3p inhibitor in U251 cells and the impact of hypoxia (24 h) on miR-584-3p expression were examined by quantitative real-time PCR. **p < 0.01, *p < 0.05 by one-way ANOVA and Student's *t*-test for miR-584-3p-i versus control. (**B**) Cell viability assay of U251 cells transfected with the miR-584-3p inhibitor. (**C**) and (**G**) Wound-healing assay of miR-584-3p inhibitor-transfected U251 cells. At 48 h after transfection, a wound was formed by scraping, and the wound size was measured again after 12 h. (**D** and **G**) Wound-healing assay under hypoxic conditions for 12 h. (**E** and **H**) The pro-migratory effect of the miR-584-3p inhibitor on U251 cell migration was examined by Transwell migration assays. At 48 h after transfection, a cell suspension was added to the upper chamber of an uncoated Transwell membrane insert, and the lower chamber was filled with medium. Cells were cultured under normoxic or hypoxic conditions for 24 h. Then, migratory cells were stained, and the average number of cells was counted in triplicate. (**F** and **I**) The pro-invasive effect of the miR-584-3p inhibitor on U251 cell invasion was examined by Matrigel invasion assays. At 48 h after transfection, a cell suspension was added to the upper chamber of a 1:4 BD Matrigel-coated Transwell membrane insert, and the lower chamber was filled with medium. Cells were cultured under normoxic or hypoxic conditions for 24 h. Then, invasive cells were stained, and the average number of cells was counted in triplicate. *p < 0.01 by one-way ANOVA and Student's *t*-test for normoxia with miR-584-3p inhibitor versus control and hypoxia control versusnormoxia control. *p < 0.01 by one-way ANOVA and Student's *t*-test for hypoxia with miR-584-3p inhibitor versus control.

Hypoxia is known to play an important role in tumor progression. In particular, hypoxia has been shown to promote cellular migration in glioma [37]. To investigate whether miR-584-3p down-regulation stimulates hypoxia-mediated glioma cell migration, we used a miR-584-3p inhibitor. miRNA inhibitors are synthetic, 2′-O-methyl-modified, single-stranded molecules that interfere with miRNA function by sequestering them via irreversible binding [38, 39]. To determine the knockdown efficiency of the miR-584-3p inhibitor, miR-584-3p expression was examined by real-time PCR (Figure 2A). The results were consistent with our previous findings, showing that hypoxia promoted the up-regulation of miR-584-3p expression, and they also confirmed the high efficiency of the miR-584-3p inhibitor. As a proof of principle, the transient transfection of 80 nM inhibitor significantly reduced endogenous miR-584-3p expression (Figure 2A) without affecting glioma cell viability (Figure 2B). Surprisingly, we observed a significant and consistent change in the pro-migratory effects of the miR-584-3p inhibitor under various conditions, regardless of the oxygen level. A wound-healing assay performed using U251 and U87 glioma cells revealed that the miR-584-3p inhibitor significantly promoted their migration, even under normoxic conditions (Figure 2C, 2G, Figure 4A), and evidently potentiated the hypoxia-induced pro-migratory effects (Figure 2D, 2G, Figure 4B).

**Figure 3:**
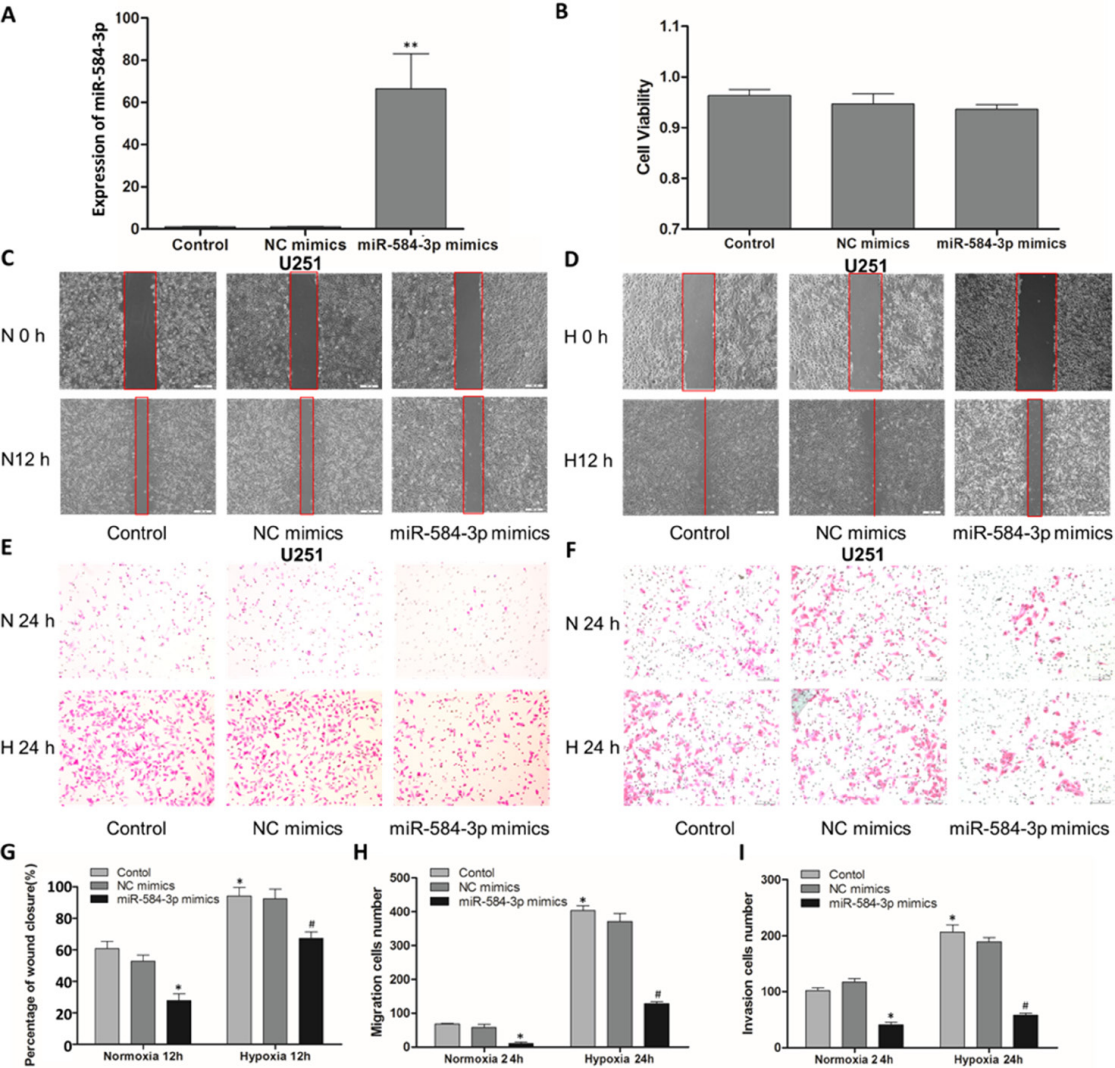
miR-584-3p overexpression suppressed the migratory and invasive capacities of human glioma cells (**A**) The overexpression efficiency of miR-584-3p mimics in U251 cells and the impact of hypoxia (24 h) on miR-584-3p expression were examined by quantitative real-time PCR. **p < 0.01 by unpaired Student's *t*-test for miR-584-3p mimics versus control. (**B**) Cell viability assay of U251 cells transfected with the miR-584-3p mimics. (**C** and **G**) Wound-healing assay of miR-584-3p mimic-transfected U251 cells. At 48 h after transfection, a wound was formed by scraping, and the wound was measured again after 12 h. (**D** and **G**) Wound-healing assay under hypoxic conditions for 12 h. (**E** and **H**) The anti-migratory effect of the miR-584-3p mimics on U251 cell migration was examined by Transwell migration assays. At 48 h after transfection, a cell suspension was added to the upper chamber of an uncoated Transwell membrane insert, and the lower chamber was filled with medium. Cells were cultured under normoxic or hypoxic conditions for 24 h. Then, migratory cells were stained, and the average number of cells was counted in triplicate. (**F** and **I**) The anti-invasive effect of the miR-584-3p mimics on U251 cell invasion was examined by Matrigel invasion assays. At 48 h after transfection, a cell suspension was added to the upper chamber of a 1:4 BD Matrigel-coated Transwell membrane insert, and the lower chamber was filled with medium. Cells were cultured under normoxic or hypoxic conditions for 24 h. Then, invasive cells were stained, and the average number of cells was counted in triplicate. *p < 0.01 by one-way ANOVA and Student's *t*-test for normoxia with miR-584-3p mimics versus control and hypoxia control versus normoxia control. *p < 0.01 by one-way ANOVA and Student's *t*-test for hypoxia with miR-584-3p mimics versus control.

**Figure 4:**
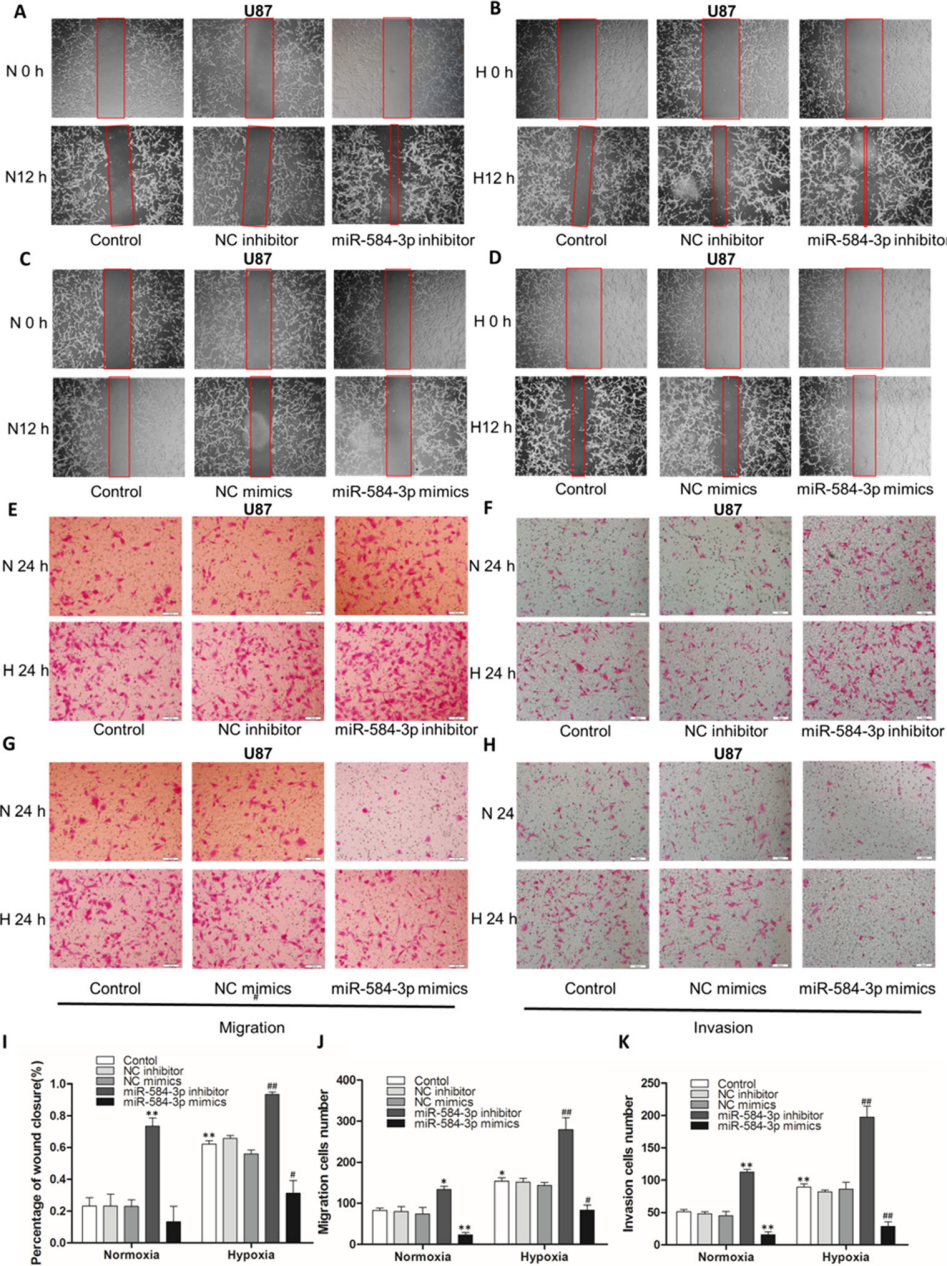
miR-584-3p knockdown enhanced the migratory and invasive capacities of human glioma cells and miR-584-3p overexpression suppressed the migratory and invasive capacities of human U87 glioma cells (**A**) Wound-healing assay of miR-584-3p inhibitor-transfected U87 cells. At 48 h after transfection, a wound was formed by scraping, and the wound was measured again after 12 h. (**B**) Wound-healing assay under hypoxic conditions for 12 h. (**C**) Wound-healing assay of miR-584-3p mimic-transfected U87 cells. At 48 h after transfection, a wound was formed by scraping, and the wound was measured again after 12 h. (**D**) Wound-healing assay under hypoxic conditions for 12 h. (**E**) The pro-migratory effect of the miR-584-3p inhibitor on U87 cell migration was examined by Transwell migration assays. At 48 h after transfection, a cell suspension was added to the upper chamber of an uncoated Transwell membrane insert, and the lower chamber was filled with medium. Cells were cultured under normoxic or hypoxic conditions for 24 h. Then, migratory cells were stained, and the average number of cells was counted in triplicate. (**F**) The pro-invasive effect of the miR-584-3p inhibitor on U87 cell invasion was examined by Matrigel invasion assays. At 48 h after transfection, a cell suspension was added to the upper chamber of a 1:4 BD Matrigel-coated Transwell membrane insert, and the lower chamber was filled with medium. Cells were cultured under normoxic or hypoxic conditions for 24 h. Then, invasive cells were stained, and the average number of cells was counted in triplicate. (**G** and **H**) The anti-migratory effect of the miR-584-3p mimics on U87 cells was examined by Transwell migration and Matrigel invasion assays. (**I** to **K**) Statistical graphs for wound-healing, Transwell migration and Matrigel invasion assays of U87 cells. The graphs show the mean ± SD and *p < 0.01 and **p < 0.001, as determined by Student's *t*-test, for groups versus normoxia control and *p < 0.01, ^##^*p* < 0.001 for groups versus hypoxia control.

To further investigate the pro-migratory effects of the miR-584-3p inhibitor, we examined U251 and U87 glioma cells migration using Transwell migration assays. Consistent with the wound-healing assay results, the miR-584-3p inhibitor exerted a powerful effect on glioma cell migration (Figure 3E, 3H, Figure 4E). This finding was of particular concern because hypoxia remains one of the most detrimental conditions for malignant human glioma. The synergetic pro-migratory effects of the miR-584-3p inhibitor under hypoxia had dramatic consequences *in vitro* (Figure 3E, 3H), suggesting that these effects of miR-584-3p deficiency were most likely related to the poorer prognosis of the patients with high-grade (III–IV) glioma and a low miR-584-3p expression level (Figure 1F, 1G).

Furthermore, we observed similar results (Figure 3F, 3I, Figure 4F) for Matrigel invasion assays. Taken together, our results clearly demonstrate that miR-584-3p knockdown markedly promotes the migratory and invasive capacities of human glioma cells and aggravates the hypoxia-induced pro-migratory and pro-invasive effects.

### miR-584-3p antagonizes the hypoxia-induced pro-migratory and pro-invasive effects on human glioma cells

To understand the mechanisms involved in the tumor suppressive function of miR-584-3p, we investigated the effects of miR-584-3p overexpression using miRNA mimics. Mimics are synthetic, double-stranded, modified RNA molecules that imitate the functions of endogenous miRNAs [40]. First, we validated that the transient transfection of 80 nM miR-584-3p mimic into human U251 glioma cells resulted in the overexpression of miR-584-3p by over 50-fold (Figure 3A) without affecting glioma cell viability (Figure 3B). Then, as expected, we observed a significant and consistent change in the anti-migratory effect of miR-584-3p, particularly under hypoxic conditions. The wound-healing assay revealed that miR-584-3p overexpression significantly inhibited U251 and U87 glioma cell migration under normoxic conditions (Figure 3C, 3G, Figure 4C) and that it strongly antagonized the hypoxia-induced pro-migratory effects (Figure 3D, 3G, Figure 4D).

Further Transwell migration assays of U251 and U87 cells also showed an anti-migratory effect of miR-584-3p (Figure 3E, 3H, Figure 4G, 4H). Moreover, miR-584-3p overexpression significantly inhibited glioma cell migration under hypoxic conditions (Figure 3E, 3H, Figure 4G). Similar results were observed using Matrigel invasion assays (Figure 3F, 3I, Figure 4H). Thus, miR-584-3p overexpression clearly inhibited the migratory and invasive capacities of human glioma cells and strongly antagonized the hypoxia-induced pro-migratory and pro-invasive effects.

These results and our preliminary observations that the subgroup of high-grade (III–IV) glioma patients with high miR-584-3p expression had a significantly prolonged postoperative survival time (Figure 1G) suggest that miR-584-3p could be a valuable tool for the development of new anti-invasive therapeutic strategies for glioma.

In contrast, we observed that miR-584-5p had no effect on the hypoxia-induced migration or invasion of U251 glioma cells (Figure S2). These findings further indicated that miR-584-3p and miR-584-5p were completely different mature miRNAs originating from miR-584 pre-miRNA. All of the above results were also similarly demonstrated in A172 and T98G glioma cells (data not shown).

### miR-584-3p inhibits hypoxia-induced stress fiber formation

**Figure 5:**
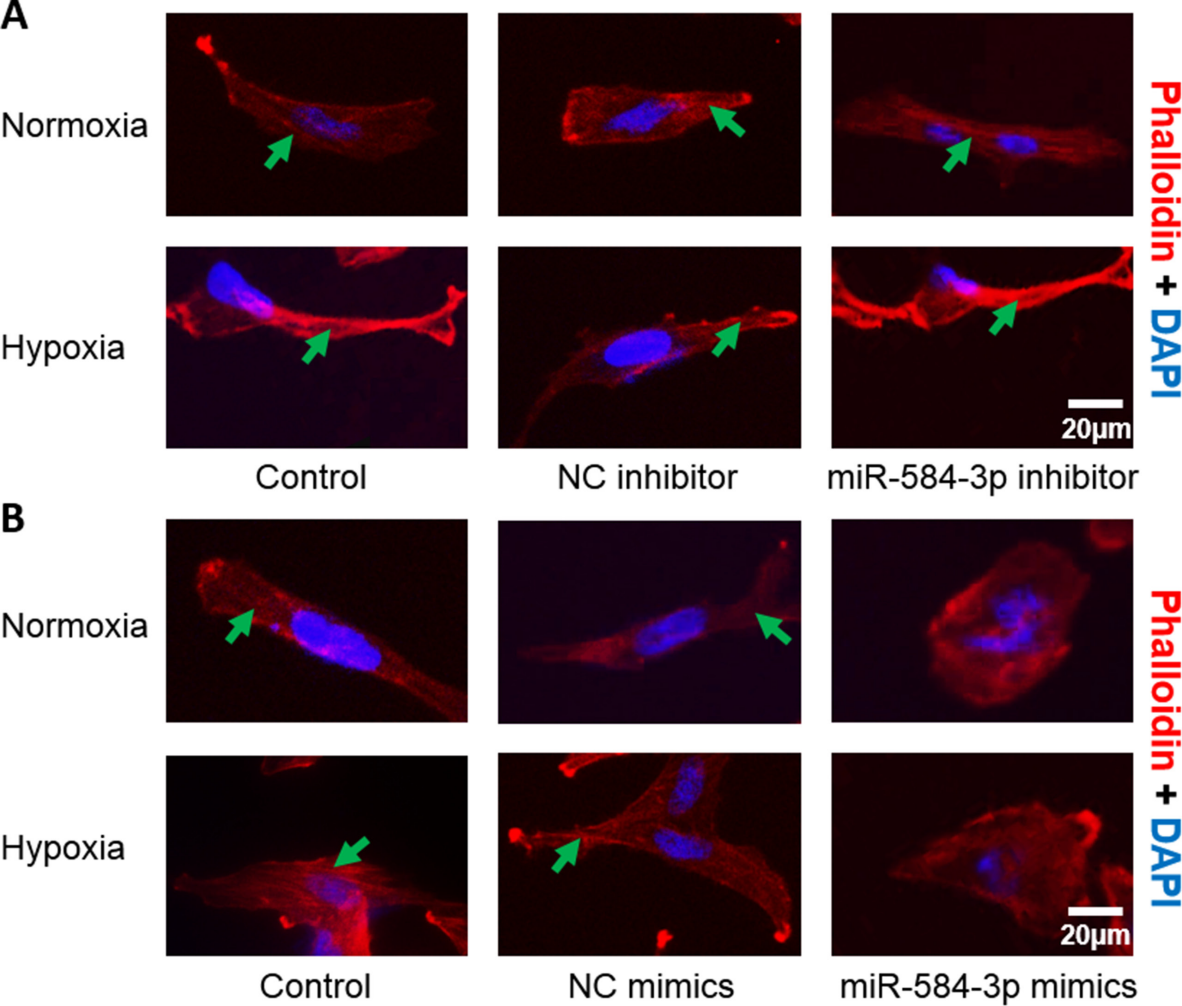
Evaluation of the effects of miR-584-3p on cytoskeletal re-organization in transfected U251 cells (**A**) Effect of miR-584-3p inhibitor transfection on U251 cell stress fiber formation under normoxic and hypoxic conditions. Cells were fixed and stained with Texas Red phalloidin and DAPI as described in the Materials and methods section. The data are representative of three independent experiments. The green arrows indicate stress fibers. (**B**) Effect of miR-584-3p mimic transfection on U251 cell stress fiber formation under normoxic and hypoxic conditions. After cells were transfected and incubated, they were fixed and stained with Texas Red phalloidin and DAPI as described in the Materials and methods section. The data are representative of three independent experiments. The green arrows indicate stress fibers.

In recent years, accumulating evidence has demonstrated the pro-migratory and pro-invasive effects of hypoxia. In this regard, we have previously shown that exposing glioma cells to hypoxic conditions enhances their motility. To determine the effect of miR-584-3p on motility-related morphological changes, we transiently transfected U251 glioma cells with the miR-584-3p inhibitor or mimics and then subjected these cells to hypoxic conditions for 12 h. We then stained them to visualize the stress fibers using phalloidin, as described in the Materials and methods section. Stress fiber formation involves cytoskeletal re-organization, as mediated byRhoA/ROCK pathway activation [41]. Our results showed that miR-584-3p knockdown facilitated stress fiber formation (Figure 5A) and that its overexpression impaired hypoxia-induced stress fiber formation (Figure 5B). The ability of miR-584-3p to inhibit actin stress fiber formation, an evident cytoskeletal change that is critical to cell motility, suggests that it may hinder the migratory and invasive capacities of glioma cells and may suppress the formation of actin stress fibers by modulating the RhoA/ROCK pathway.

### miR-584-3p antagonizes the hypoxia-induced pro-migratory and pro-invasive effects by targeting ROCK-1

**Figure 6:**
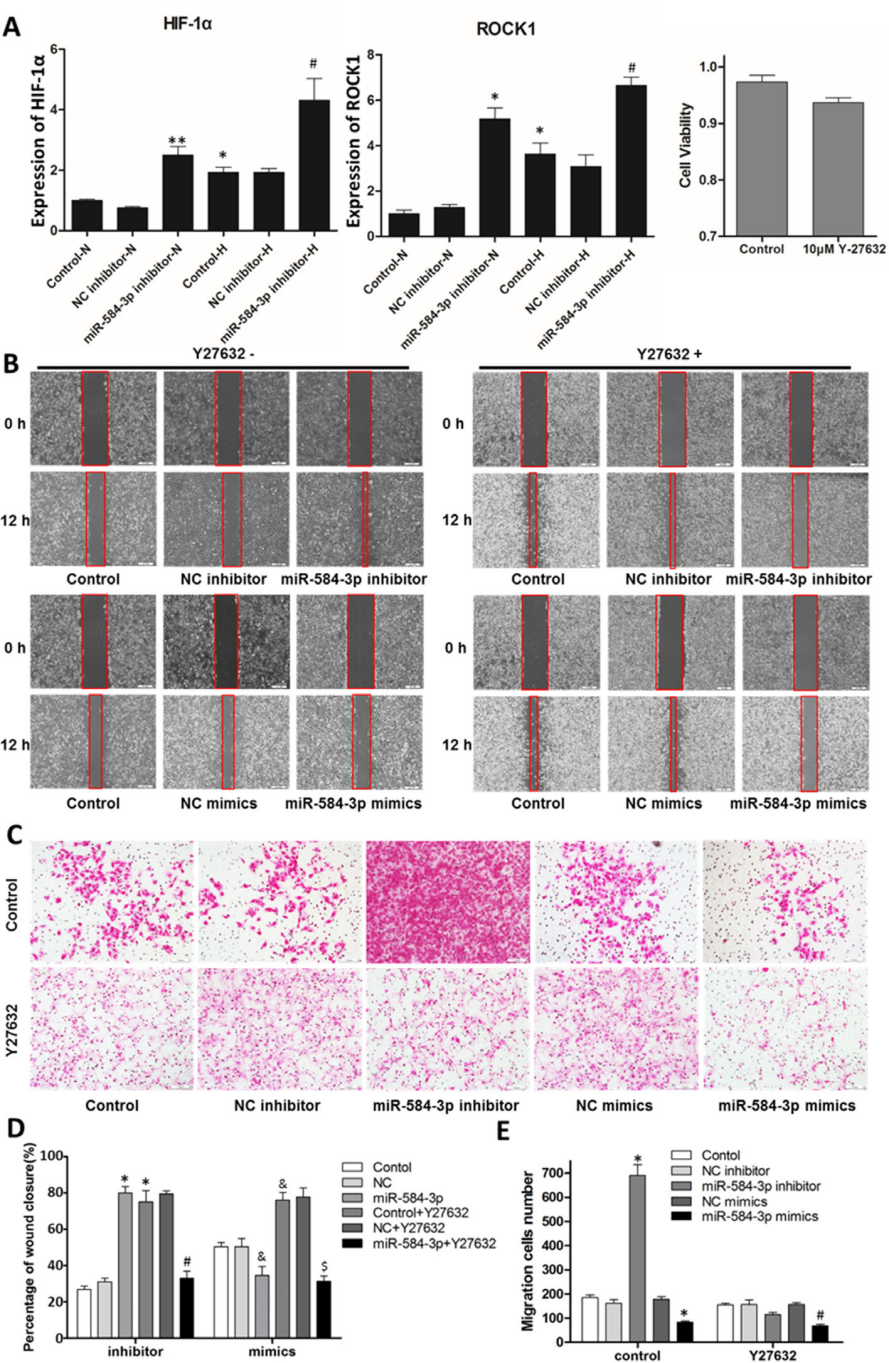
Specific inhibition of ROCK1 by Y-27632 blocked the pro-migratory effect of the miR-584-3p inhibitor (**A**) The expression of HIF-1α and ROCK1 after miR-584-3p knockdown in U251 cells and the impact of hypoxia (24 h) were examined by quantitative real-time PCR. The right panel shows the results of cell viability assay of U251 cells treated with 10 μM Y-27632 for 12 h. *p < 0.01, **p < 0.001, as determined by Student's *t*-test, for groups versus normoxia control and *p < 0.01 for groups versus hypoxia control. (**B** and **D**) Wound-healing assays of the miR-584-3p inhibitor- and mimic-transfected U251 cells treated with Y-27632. At 36 h after transfection, U251 glioma cells were treated with 10 μM Y-27632 for 12 h, and then a wound was created by scraping. The wound was measured again after 12 h. *p < 0.01, as determined by Student's *t*-test, for groups versus inhibitor control, *p < 0.01 for groups versus inhibitor Y27632 control, ^&^*p* < 0.01 for groups versus mimic control, and *p < 0.01 for groups versus mimic Y27632 control. (**C** and **E**) Effects of Y-27632 on the miR-584-3p-inhibitor- and mimic- transfected U251 cells were examined by Transwell migration assays. At 36 h after transfection, U251 glioma cells were treated with 10 μM Y-27632 for 12 h. Then, the cell suspension was added to the upper chamber of an uncoated Transwell membrane insert, the lower chamber was filled with medium, and the cells were cultured for 12 h. Finally, migratory cells were stained, and the average number of cells was counted in triplicate. The graph shows the mean ± SD and *p < 0.01, as determined by Student's *t*-test, for groups versus control, and *p < 0.01 for groups versus Y27632 control.

To investigate whether miR-584-3p and the RhoA/ROCK pathway are linked, we utilized a whole-genome mRNA gene expression array, as mentioned previously, to compare the expression levels of RhoA/ROCK pathway members between normoxic and hypoxic U251 cells (data not shown). The microarray results suggested that ROCK-1 was the most likely target of miR-584-3p. We further examined this finding using quantitative real-time PCR (Figure 6A), and the results supported the notion that miR-584-3p knockdown induces ROCK-1 expression. In addition, the effects of hypoxic conditions on HIF-1α and ROCK-1 expression were examined (Figure 6A). Further, we assessed whether specific inhibition of the Rho-ROCK pathway using the ROCK1-specific inhibitor Y-27632 or ROCK1-specific small interfering RNA would block the pro-migratory effect of the miR-584-3p inhibitor. U251 and U87 glioma cells were treated with Y-27632 and transfected with ROCK1 siRNA, respectively. Following treatment with Y-27632 for 12 h, wound-healing assay was performed on U251 cells, revealing that the pro-migratory effect of the miR-584-3p inhibitor was significantly suppressed by ROCK1 inhibition (Figure 6B upper and 6D left). The anti-migratory effect of miR-584-3p overexpression was slightly enhanced by Y-27632, with no significant difference compared with untreated cells (Figure 6B lower and 6D right). Further Transwell migration assays also showed that Y27632 treatment resulted in the consistent and significant suppression of the pro-migratory effect of the miR-584-3p inhibitor and that it had an indistinguishable synergistic effect with miR-584-3p mimics (Figure 6C, 6E).

**Figure 7:**
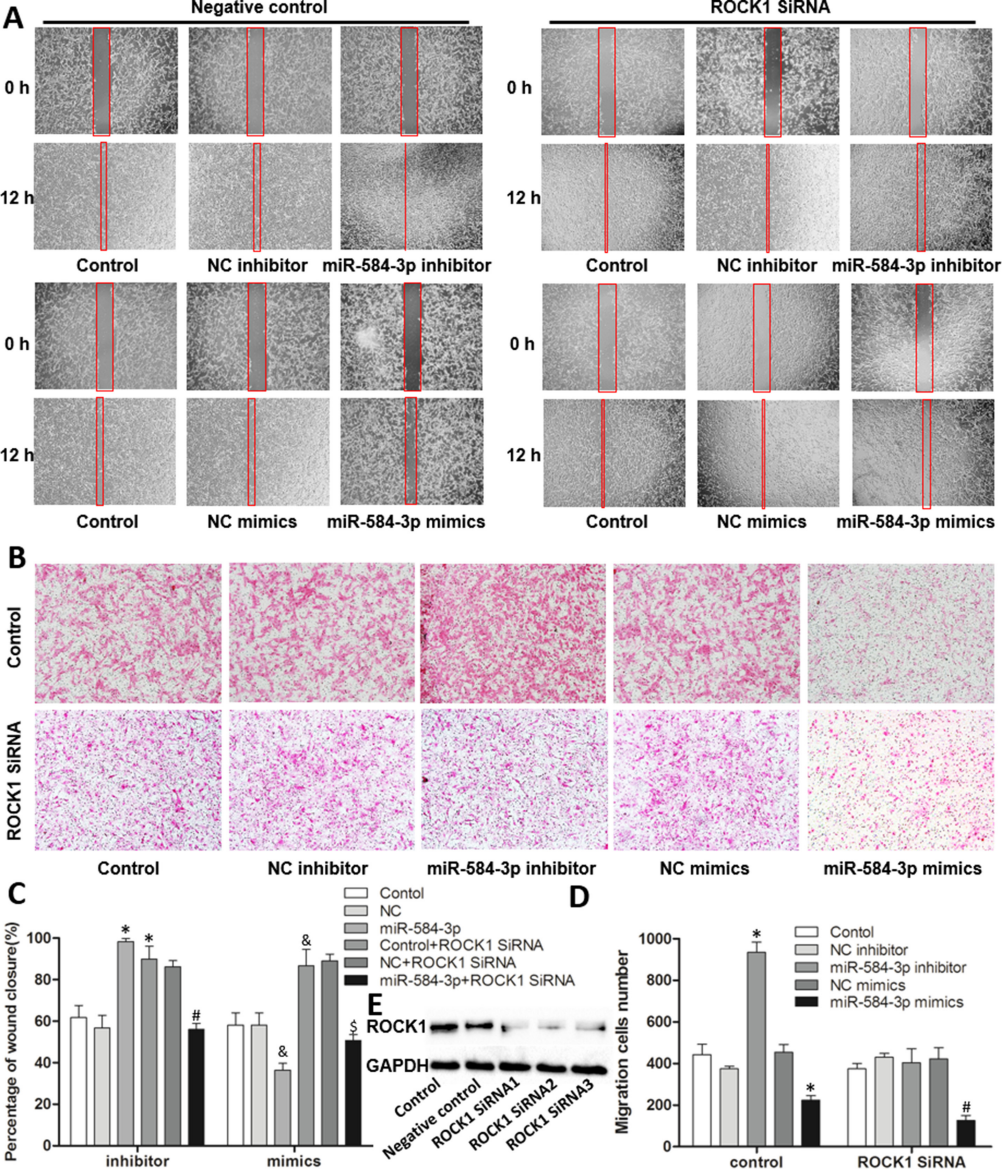
Specific siRNA-mediated inhibition of ROCK1 blocked the pro-migratory effect of miR-584-3p inhibition (**A** and **C**) Wound-healing assays of miR-584-3p inhibitor- and mimic-transfected U87 cells co-transfected with ROCK1 siRNA. At 24 h after co-transfection, U87 glioma cells were wounded by scraping. The wound was measured again after 12 h. *p < 0.01 by Student's *t*-test for groups versus inhibitor control, *p < 0.01 for groups versus inhibitor siRNA control, ^&^*p* < 0.01 for groups versus mimic control, and *p < 0.01 for groups versus mimic siRNA control. (**B** and **D**) Effects of ROCK1 siRNA on miR-584-3p-inhibitor- and mimic-transfected U87 cells were examined by Transwell migration assays. At 24 h after co-transfection, the U87 glioma cell suspension was added to the upper chamber of an uncoated Transwell membrane insert, the lower chamber was filled with medium, and the cells were cultured for 12 h. Finally, migratory cells were stained, and the average number of cells was counted in triplicate. The graph shows mean ± SD and *p < 0.01, as determined by Student's *t*-test, for groups versus control, and *p < 0.01 for groups versus siRNA control. (**E**) The ROCK1 protein levels in U87 cells transfected with ROCK1 siRNAs or negative control. At 72 h after transfection, total protein was extracted and analyzed by western blotting. GAPDH was used as a loading control.

To verify that the RhoA/ROCK1 pathway is involved in the function of miR-584-3p, we investigated the effect of ROCK1 knockdown on miR-584-3p activity. A sufficient concentration of ROCK1-specific small interfering RNAs was transfected into cultured U87 glioma cells, as demonstrated by the knockout efficiency (Figure 7E). Wound-healing and Transwell migration assays were performed at 24 h after transfection. Similar to Y-27632, ROCK1 knockdown in U87 cells completely eliminated the pro-migratory effect of the miR-584-3p inhibitor, as shown by wound-healing (Figure 7A upper and 7C left) and Transwell migration assays (Figure 7B, 7D). The anti-migratory effect of the miR-584-3p mimics was not significantly promoted by ROCK1 inhibition during wound-healing (Figure 7A lower and 7C right), but it showed a detectable significant difference in Transwell migration assays (Figure 7B, 7D). In addition, both the Y-27632 and ROCK1 siRNA treatments resulted in morphologic changes of U251 and U87 cells with threadlike pseudopodia (Figures 6 and 7), as described in other studies, and the pro-migratory effect of ROCK1 inhibition itself was only observed in wound-healing assays (Figures 6B, 6D, and 7A, 7C); however, Transwell migration assays were more representative of the *in vivo* conditions (Figures 6C, 6E, and 7B, 7D). The data for the Y-27632-treated U87 cells and ROCK1 siRNA-transfected U251 cells are not shown.

**Figure 8:**
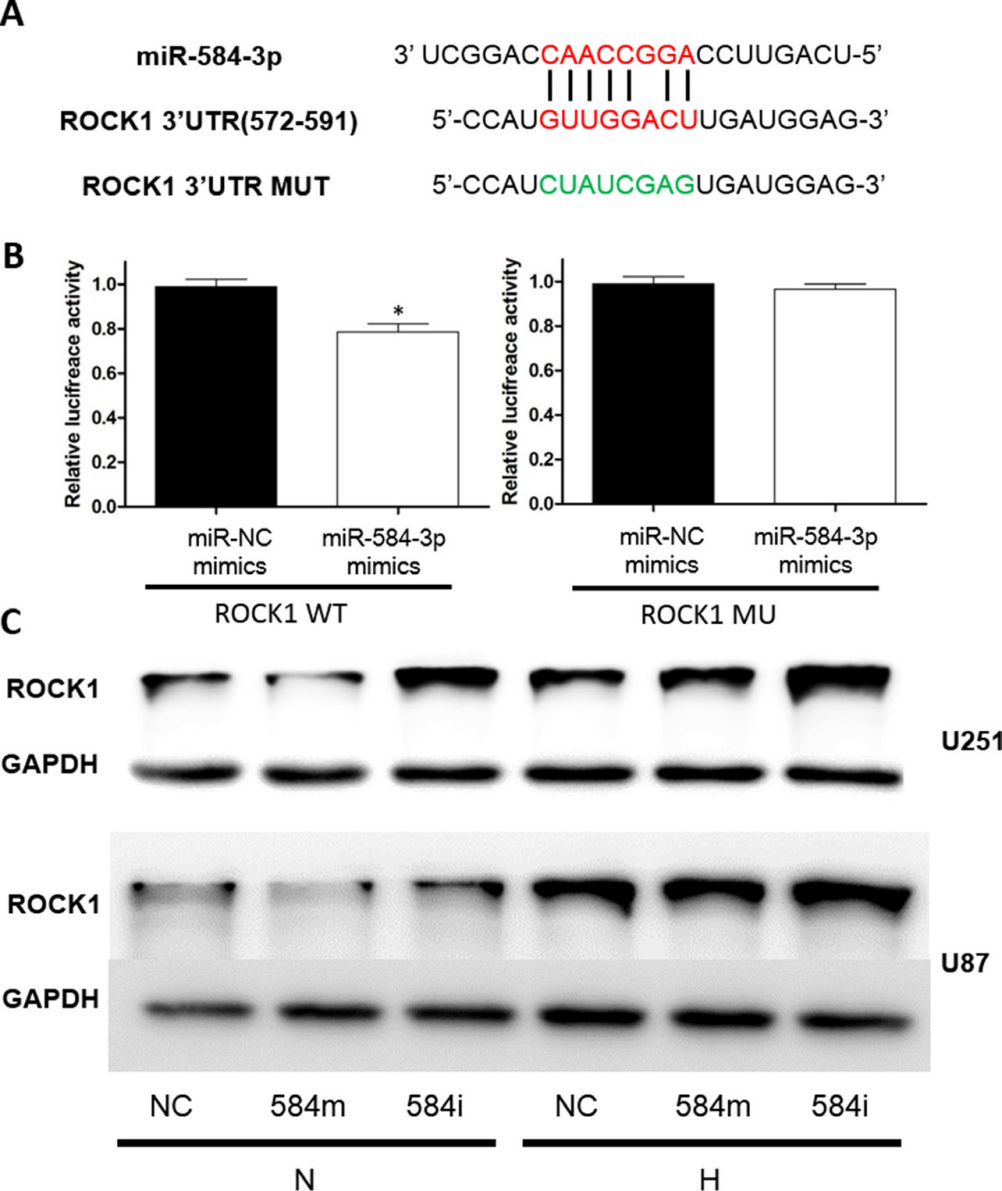
ROCK1 is a target gene of miR-584-3p (**A**) Sequence of the miR-584-3p binding site in ROCK1 and (**B**) results of 3′-UTR luciferase assay. The miR-584 binding site in the ROCK1 3′-UTR was predicted using bioinformatics. A 3′-UTR vector and miR-584-3p or miRNA negative control were co-transfected into U251 cells. Relative luciferase activity was measured in cell lysates at 48 h after transfection. The luciferase activity levels were compared with those of the miRNA negative control-transfected cells, which were normalized to 1. (**C**) The ROCK1 protein levels in U251 and U87 cells transfected with miR-584-3p. U251 and U87 cells were transfected with miR-584-3p or negative control miRNA. At 72 h after transfection, total protein was extracted and analyzed by western blotting. GAPDH was used as a loading control.

Second, we searched for the target oncogenes of the tumor suppressor miR-584-3p in PITA (Segal Lab of Computational Biology) and identified a potential target oncogene, ROCK-1. To assess the direct inhibitory effect of miR-584-3p on ROCK-1 gene transcription, 3′-UTR seed sequence mutation and 3′-UTR luciferase assays were performed using U251 glioma cells (Figure 8A). The luciferase activity in miR-584-3p-transfected cells decreased to approximately 78.6% of that observed using control miRNA (Figure 8B). To examine the inhibitory effect of miR-584-3p at the protein level, we performed western blot analysis at 48 h after miR-584-3p inhibitor and mimic transfection into U251 and U87 cells. We observed that the ROCK-1 protein levels were significantly up-regulated by hypoxia treatment in both U251 and U87 cells and that its levels in the miR-584-3p mimic-transfected glioma cells under normoxia decreased significantly compared with those in the negative control miRNA-transfected glioma cells. Surprisingly, miR-584-3p overexpression failed to efficiently decrease the hypoxic ROCK1 protein level. In contrast, its protein level increased significantly in the miR-584-3p inhibitor-transfected glioma cells, particularly under hypoxic conditions (Figure 8C). Although the miR-584-3p inhibitor also increased the transcriptional level of HIF-1α (Figure 6A), further protein examination found no differences (data not shown).

### Negative correlation of miR-584-3p expression with the ROCK-1 level in tumor tissues from high-grade (III–IV) glioma patients

To examine whether miR-584-3p expression is correlated with the ROCK-1 level in human glioma, we detected ROCK-1 expression in 26 human glioma specimens of different grades by immunohistochemical staining. As shown in Figure 8A and 8C, miR-584-3p expression was strongly negatively correlated with the ROCK-1 level in the tumor tissues from the high-grade (III–IV) glioma patients. We also found that the ROCK-1 level was negatively correlated with the survival time of the glioma patients (Figure 8A). Consistent with this finding, miR-584-3p expression was also associated with the survival time of the glioma patients (Figure 1G). Further immunohistochemical staining of the tissues from the low-grade (I–II) glioma patients also showed that the expression levels of both miR-584-3p and ROCK-1 were low in the low-grade glioma tissues (Figure 8B, Figure 1F). Taken together, these results demonstrate that the miR-584-3p level is positively correlated with the survival time of high-grade (III–IV) glioma patients and that it acts as a prognostic biomarker by targeting ROCK-1.

### miR-584-3p antagonizes the hypoxia-induced pro-invasive effects in an orthotopically xenografted glioma mouse model

To verify that inhibition of the hypoxia-induced invasive effect of miR-584-3p on glioma cells occurs *in vivo*, lentivirus vector-transfected U87 cells were transplanted into nude mouse brains through intracranial injection. We adopted a noninvasive and harmless method to continually observe the sizes of the intracranial tumors in the model mice by T2-weighted magnetic resonance imaging (T2WI-MRI). The results showed that the invasiveness of the defective miR-584-3p-expressing tumor over a two-month period significantly increased but that miR-584-3p overexpression did not strongly reduce the extent of glioma (Figure 10A, 10C). Hematoxylin and eosin (HE) staining confirmed the extremely aggressive status of the miR-584-3p knockout xenografted glioma relative to the control group. miR-584-3p overexpression failed to further strongly inhibit the invasion of glioma cells (Figure 10B). Further immunohistochemical staining also showed the negative relationship between the miR-584-3p and ROCK-1 levels (Figure 10E). However, the survival prognosis of the miR-584-3p-overexpressing group was improved (Figure 10D). Therefore, miR-584-3p deficiency undoubtedly promoted the full invasive capacities of the glioma cells from the mouse models. These findings are highly consistent with the poor clinical survival prognosis of the patients with low miR-584-3p expression, as shown in Figure 1G.

**Figure 9:**
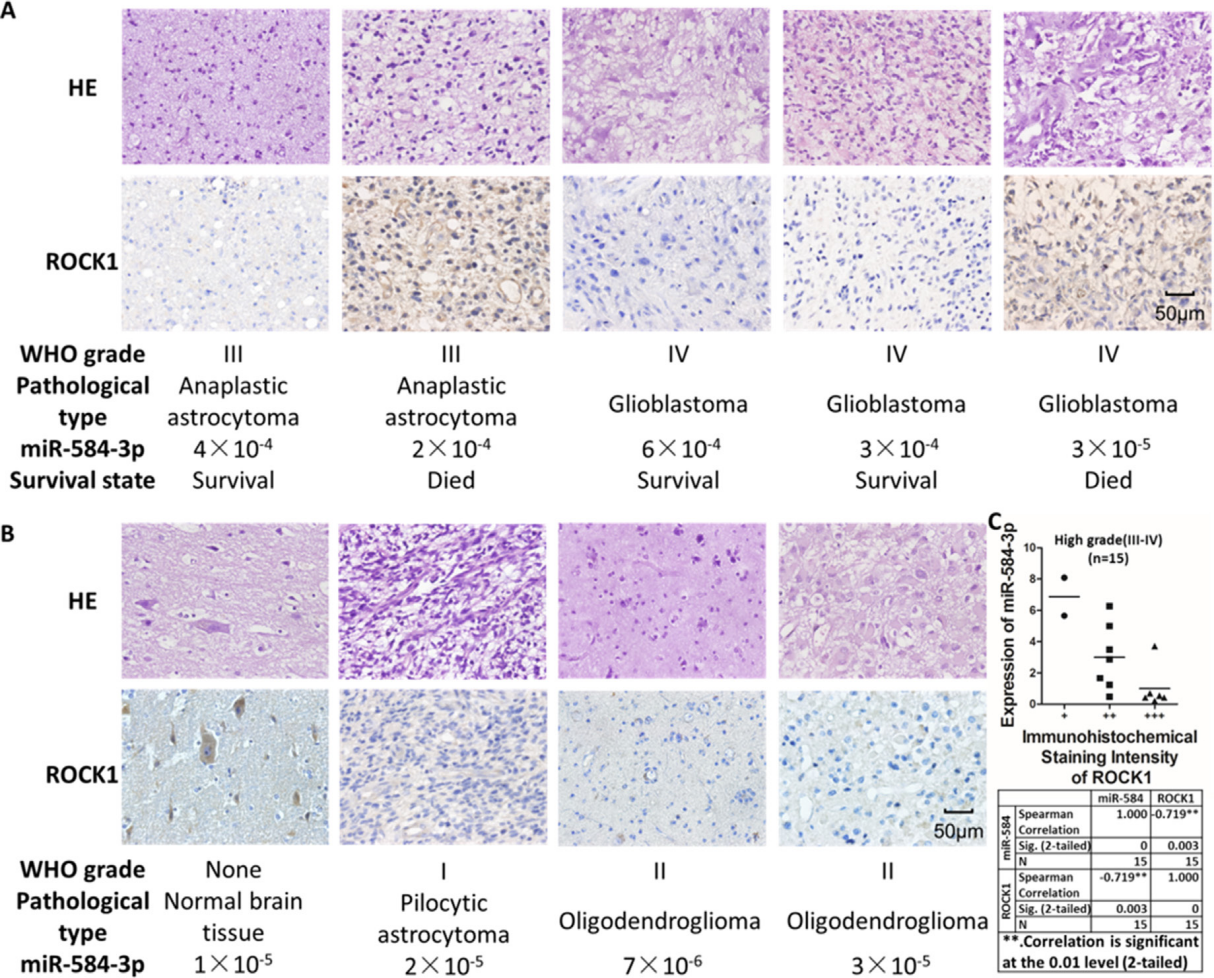
The miR-584-3p level is negatively correlated with ROCK-1 in tumor tissues of high-grade (III–IV) glioma patients (**A**) The expression of ROCK-1 in high-grade glioma was determined by immunohistochemical staining. (**B**) The immunohistochemical staining of ROCK-1 in low-grade glioma and normal brain tissues. (**C**) The miR-584-3p level is negatively correlated with the ROCK-1 level in tumor tissues of high-grade (III–IV) glioma patients. Spearman's correlation coefficient was −0.719**p = 0.003, and **correlation was significant at the 0.01 level (2-tailed).

**Figure 10:**
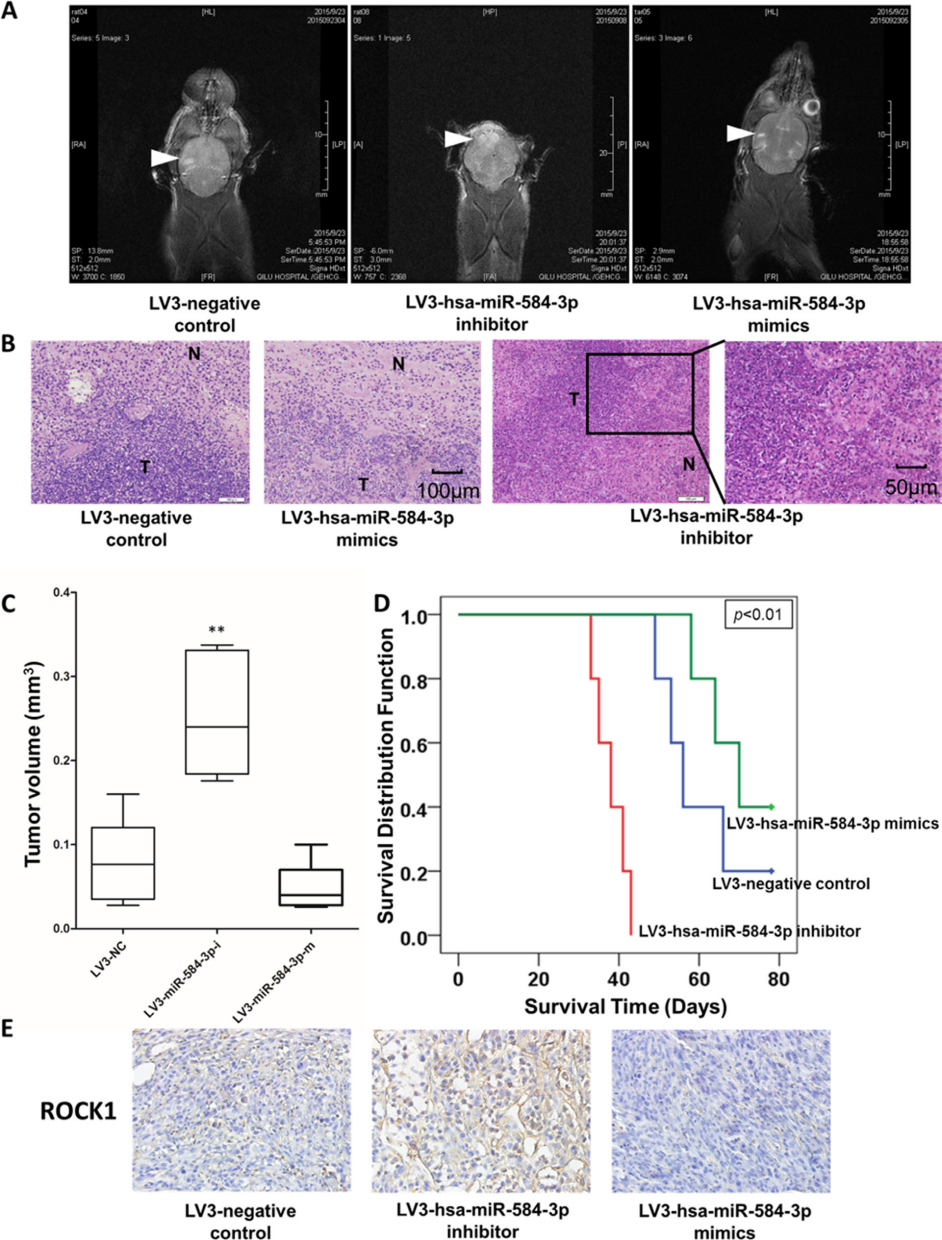
miR-584-3p antagonizes hypoxia-induced pro-invasive effects in an orthotopically xenografted glioma mouse model (**A**) T2-weighted magnetic resonance imaging (T2WI-MRI) of the sizes of the intracranial tumors in the model mice on day 60. (**B**) Hematoxylin and eosin (H & E) staining of orthotopically xenografted glioma. (**C**) The tumor volume on day 60, as determined by MRI measurement. (**D**) Prognostic significance of the model mice. (**E**) Immunohistochemical staining of ROCK-1 in orthotopically xenografted glioma.

## DISCUSSION

In recent years, the expression of tumor-suppressive miRNAs in glioma has been a topic of interest for antineoplastic research, and accumulating evidence has demonstrated the potentials of these antineoplastic miRNAs as prognostic indicators and therapeutic targets [42]. Considering that miRNA research has advanced from the identification of an initial association with glioma to the commercial development of miRNA-based therapeutics in less than a decade, the anticipation of significant developments in this field with the ultimate improvement of patient outcomes is reasonable [43]. Several recent reports have confirmed that numerous highly expressed miRNAs, such as miR-10b [27], miR-21 [44–46], miR-210 [47], and miR-221/222 [18], are predictive of poor prognosis in glioma patients. However, an increasing number of tumor-suppressive miRNAs have also been discovered, including miR-637 [48], miR-663 [49], miR-218 [30], miR-128 [50], and miR-34a [51]. Here, we identified miR-584-3p as a novel tumor-suppressive miRNA. Although a few well-designed studies of miR-584 have demonstrated its role as a tumor suppressor in glioma [34], renal cell carcinoma [35], and breast cancer [36], all of these studies have considered miR-584-5p to be the unique mature miRNA originating from miR-584 pre-miRNA and have completely ignored the other completely different mature miRNA originating from this pre-miRNA. In the present work, we outlined the role of the novel tumor-suppressive miR-584-3p, in spite of its lower copy number compared with miR-584-5p. Importantly, our findings are the first to show an unexpected role of miR-584-3p in preventing glioma cell invasion rather than miR-584-5p, as well as the prognostic value of miR-584-3p in malignant glioma.

Cells undergo a variety of biological responses when subjected to hypoxic conditions, including the activation of signaling pathways that regulate proliferation, angiogenesis, metastasis, and apoptosis. Tumor cells have adapted these pathways to survive and even grow under hypoxic conditions. Tumor hypoxia is associated with poor prognosis and resistance to anti-tumor therapy [52]. Brain gliomas, particularly the highly aggressive GBM subtype with its necrotic tissues, are affected similarly by hypoxia. Additionally, cell invasion, apoptosis, chemoresistance, and radiation resistance processes are affected by hypoxia. The extent of the influence of hypoxia on these processes makes it an attractive therapeutic strategy for glioma [53]. In surgical specimens of human GBM, cells surrounding necrotic areas appear to migrate away from them [54, 55], possibly implicating hypoxia in the regulation of this process. This idea has been further supported by studies of both *in vitro* and *in vivo* models suggesting that tumor hypoxia results in increased GBM cell migration and invasion capacities [56]. Recent reports have identified various miRNAs that may be involved in this process [45, 46]. Therefore, using a miRNA microarray, we compared the changes in the miRNA expression profiles between normoxic and hypoxic conditions in glioma cells. Then, we selected miR-584-3p as a new potential tumor suppressor candidate based on its significant up-regulation in hypoxic glioma cells. No previous reports of the role of miR-584-3p in glioma exist. We also performed real-time PCR analyses using U251 and U87 cells and surgically removed glioma tissues from 26 patients to validate the microarray data. We found that miR-584-3p expression was significantly higher in the high-grade (III–IV) glioma patients than in the low-grade (I–II) glioma patients. However, surprisingly, the subgroup of patients with higher miR-584-3p expression among the high-grade (III–IV) glioma patients presented a significantly prolonged postoperative survival time. Collectively, these findings demonstrate that miR-584-3p acts as a tumor suppressor and that it represents a potential prognostic biomarker of malignant glioma. Thus far, the role of miR-584-3p in tumors, including gliomas, has not been reported.

The pathways implicated in the tumor suppressive role of miR-584-3p are poorly defined. Therefore, we sought to determine the relationship between this miRNA and hypoxia. Based on our results and those of previous reports, we hypothesized that miR-584-3p could antagonize hypoxia-induced glioma malignant progression and might act by inhibiting migration and invasion. To test this hypothesis, we performed functional analyses (proliferation, invasion, migration, apoptosis, and cell cycle assays (data not shown)) of miR-584-3p using miR-584-3p-transfected cells. As expected, cell motility dramatically decreased after miR-584-3p mimic transfection, while miR-584-3p knockdown dramatically enhanced the migratory and invasive capacities of human glioma cells. Importantly, these effects of miR-584-3 were more pronounced under hypoxic conditions. Our results suggest that miR-584-3p is up-regulated in response to hypoxic stimulation and that it restricts the migratory capacities of glioma cells. Knockout of miR-584-3p promotes the full migratory capacities of glioma cells and likely enhances tumor progression. These findings may explain the poor prognosis of glioma patients with defective miR-584-3p expression. miR-584-3p may be involved in protective compensatory mechanisms and play an important role in inhibiting the hypoxia-induced invasion and migration capacities of glioma cells. Notably, low-grade glioma is associated with much less hypoxic conditions [57] that are insufficient to up-regulate miR-584-3p. Therefore, we searched for motility-related genes as potential target oncogenes downstream of miR-584-3p. Initially, we stained cells with the cytoskeleton marker phalloidin and discovered a defect in F-actin stress fiber formation after miR-584-3p overexpression. Because stress fiber formation is a cytoskeletal re-organization process mediated by RhoA/ROCK pathway activation [41], in addition to the identification of ROCK-1 as a hypoxia-induced gene in our mRNA microarray, we validated the expression of this potential target molecule by real-time PCR and examined its functional role using the ROCK-1-specific inhibitor Y27632. This inhibitor blocked the pro-migratory effect of the miR-584-3p inhibitor, as expected. Subsequently, we identified the miR-584-3p target seed sequence in the 3′-UTR region of the ROCK-1 mRNA using an online miRNA prediction tool of the Segal Lab. Next, a 3′-UTR luciferase assay was performed. The luciferase activity was significantly decreased in miR-584-3p-transfected cells compared with miRNA control-transfected cells. In addition, the ROCK-1 protein levels were significantly decreased in miR-584-3p mimic-transfected U251 and U87 cells and were elevated in miR-584-3p inhibitor-transfected glioma cells, particularly under hypoxic conditions. These results demonstrate that ROCK-1 is a direct target of miR-584-3p.

The overexpression of ROCK-1, which is induced by activated RhoA, is related to brain tumor metastasis [58]. Our present findings have shown that ROCK-1 is a miR-584-3p target gene and that miR-584-3p expression is correlated with the survival prognosis of glioma patients, suggesting that miR-584-3p may be involved in glioma progression. The mechanism by which miR-584-3p functions is summarized as follows. First, it down-regulates ROCK-1 by directly binding to the 3′-UTR region, thus inhibiting RhoA/ROCK pathway-mediated stress fiber formation. Consequently, this cytoskeletal disorder leads to decreased cell motility and dramatic inhibition of human glioma cell migration and invasion. Ultimately, the reduced aggressiveness of these tumors results in the improved prognosis of glioma patients. Our data have also indicated that miR-584-3p is a hypoxia-induced tumor suppressor that antagonizes the pro-invasive effects produced by hypoxic conditions, consistent with our clinical observations. Indeed, the finding that miR-584-3p expression was significantly higher in the high-grade glioma tissues compared with the low-grade glioma tissues makes sense because of the more hypoxic microenvironment present in high-grade glioma due to its rapid proliferation. The subgroup of high-grade (III–IV) glioma patients with high miR-584-3p expression had a significantly prolonged postoperative survival time, most likely because of the anti-tumor activity of miR-584-3p. Further evidence was also obtained by the immunohistochemical staining of 26 human glioma specimens for ROCK-1. The finding that miR-584-3p was up-regulated by hypoxic stress and in malignant glioma was logical; however, the ROCK1 level was not decreased under hypoxic conditions. Because of the differences between *in vitro* and *in vivo* conditions, some obvious phenomena occurring *in vitro* might not have occurred *in vivo*. Our *in vivo* experiments showed that miR-584-3p overexpression in solid tumors under hypoxic conditions may not have further strongly reduced glioma cell invasiveness. However, miR-584-3p deficiency undoubtedly promoted the full invasive capacities of glioma cells in the mouse models. These findings are highly consistent with the poor clinical survival prognosis of the patients with low miR-584-3p expression, as shown in Figure 1G. In addition, other regulatory molecules of ROCK1 and target molecules of miR-584-3p must exist that are involved in this complex process of migratory change that need to be studied in depth. Obviously, an additional unknown strong stimulatory mechanism of ROCK1 exists under hypoxic conditions, and miR-584-3p overexpression is only sufficient to maintain a balance. On the other hand, other target molecules might help miR-584-3p to inhibit glioma cell migration and ROCK1 expression.

In summary, our results demonstrate the anti-migratory and anti-invasive effects of a novel ROCK-1 inhibitory miRNA, miR-584-3p, and suggest that miR-584-3p may represent a prognostic biomarker of high-grade glioma. Furthermore, miR-584-3p is a potential therapeutic target for malignant glioma, particularly for patients with WHO III–IV GBMs. However, one limitation of this study is the small number of samples included. In addition, the involvement of other key invasion-associated proteins, such as Rac1, Cdc42 [59], and MMPs [60], were not investigated. Therefore, additional studies are required to substantiate our findings.

## MATERIALS AND METHODS

### Tissue samples and cell lines

The human glioma cell lines U251 and U87 were purchased from the Chinese Academy of Sciences Cell Bank. Twenty-six human glioma tissues, including ten low-grade gliomas (two grade I and eleven grade II tumors) and sixteen high-grade gliomas (five grade III and eleven grade IV tumors), were obtained from the Department of Neurosurgery of Qilu Hospital of Shandong University. The glioma specimens were verified and classified according to the WHO Classification of Tumors by two experienced clinical pathologists. Our study was approved by the Institutional Review Board of Shandong University. Written informed consent was obtained from all patients, and the hospital ethical committee approved the experiments.

### Reagents and cell culture

All cells were cultured in DMEM supplemented with 10% FBS and maintained at 37°C with 5% CO_2_ in a humidified chamber. Y-27632 (ROCK inhibitor) was purchased from Selleck Chemicals (USA). Hypoxic conditions were induced by incubating the cells in a modular incubator chamber flushed with a gas mixture containing 1% O_2_, 5% CO_2_, and 94% N_2_ at 37°C.

### Cell transfection

A mature miR-584-3p mimic, scrambled mimic control, inhibitor, scrambled inhibitor control, and miR-584-5p mimic were designed and synthesized by RiboBio (Guangzhou, China). Cell transfections and co-transfections were performed using Lipofectamine 2000 when the cells reached 70% confluence according to the manufacturer's instructions. Untransfected cells were used as blank controls, while cells transfected with scrambled oligos were used as negative controls. Transfection efficiency was verified by quantitative real-time PCR. Forty-eight hours after transfection, the glioma cells were harvested for subsequent experiments.

### Cell viability assay

U251 and U87 cells were seeded in 96-well culture plates at a density of 3000 cells/well. Cell proliferation was analyzed at 24, 48, 72, and 96 h after transfection using a Cell Counting Kit-8. A volume of 10 μL CCK-8 solution was added to each well, and the cells were incubated for another 1 h in a humidified incubator at 37°C. Then, optical density was measured at 450 nm using a microplate reader.

### Wound-healing assay

Cells (1 × 10^5^) were seeded in 6-well plates, incubated overnight, and then transfected with a test substance or vehicle. At 90% confluency, the cell monolayer was scratched with a sterile pipette tip, and floating cells were removed with PBS. The scratched plates were cultured in DMEM containing 1% FBS. Images were captured at 0 and 12 h along the scrape line under a microscope. The results are expressed as the relative scratch width based on the distance migrated relative to the original scratched distance.

### Cell migration and invasion assays

Cell migration and invasion assays were performed using Transwell chambers measuring 6.5 mm in diameter (8-μm pore size, Corning). In total, 5 × 10^4^ transfected cells in FBS-free medium were seeded in the upper chamber of an uncoated Transwell chamber (i.e., without Matrigel (BD) for the migration assay) or of a Matrigel-coated chamber. Medium containing 10% FBS was added to the lower chamber. After 12, 24, and 48 h, cells that did not migrate or invade were removed using cotton buds. Cells that migrated to or invaded the lower surface were fixed, stained with eosin for 15 min and counted under a microscope. Five random views were used to count the cells.

### Cytoskeleton staining assay

U251 cells were plated at 8000 cells/well on climbing pieces at the bottoms of wells of 6-well plates. After the cells were transfected and exposed to hypoxia, they were fixed in 4% paraformaldehyde, permeabilized using 0.1% Triton *X*-100, and stained with Texas Red phalloidin and DAPI.

### Human miRCURY^™^ LNA array analysis

U251 cells were cultured under normoxic or hypoxic conditions (3 samples were exposed to each condition as biological replicates), and then total RNA was extracted for microarray analysis using TRIzol (Invitrogen) and an miRNeasy Mini Kit (QIAGEN), according to the manufacturers' instructions. After measuring the RNA quantities in the samples using a NanoDrop 1000, the RNA samples were labeled using a miRCURY^™^ Hy3^™^/Hy5^™^ Power Labeling Kit and hybridized to a miRCURY^™^ LNA Array (v.18.0). Following hybridization, the array was washed and then scanned using an Axon GenePix 4000B microarray scanner. Scanned images were imported into GenePix Pro 6.0 software (Axon) for grid alignment and data extraction. After normalization, significant differentially expressed miRNAs were identified through volcano plot filtering. Finally, hierarchical clustering was performed to identify the distinguishable gene expression profiles among the samples.

### Bioinformatics prediction and luciferase reporter assay

The common miR-584-3p targets predicted by computer-aided algorithms were identified using multiple target prediction programs, including TargetScan 5.2, an online miRNA prediction tool of the Segal Lab, and miRBase. Reporter constructs containing pGL3-ROCK1 and pGL3-mutROCK1 (with a mutated target seed sequence) were obtained from Bio-Asia (Jinan China). Glioma cells were co-transfected with the luciferase reporters and a test substance or vehicle using Lipofectamine 2000. Forty-eight hours after the cells were transfected, luciferase assays were performed using a luciferase assay kit according to the manufacturer's instructions.

### RNA extraction and real-time quantitative PCR

Total RNA was extracted using TRIzol Reagent according to the manufacturer's protocol. Then, total RNA (50 ng) was reverse transcribed with miRNA stem-loop RT primers or with U6 RT primers using a ReverTra Ace qPCR RT Kit according to the manufacturer's instructions to generate cDNA, and a PrimeScript 1st Strand cDNA Synthesis Kit was used to reverse transcribe HIF-1α and ROCK1. Real-time PCR was performed using a SYBR Premix Ex Taq^™^ Kit and the primers shown in Figure S1. The reactions were performed with a LightCycler 2.0 Instrument. The U6 and GAPDH expression levels were used as endogenous controls. The absolute expression levels were calculated as concentration ratios using a Roche LightCycler^®^ 2.0 system.

### Western blot analysis

Total protein was extracted using RIPA buffer containing 1% phenylmethylsulfonyl fluoride. Equal amounts of protein were loaded onto a 10% SDS-polyacrylamide gel. Next, blots were incubated with a primary antibody overnight at 4°C and then with a horseradish peroxidase-conjugated secondary antibody at room temperature for 1 h. Finally, protein bands were visualized using ECL (Millipore) and detected using an ECL detection system. The primary antibody used was directed against ROCK-1 (1:1000, Abcam), and the relative integrated density values were determined based on the GAPDH protein expression level as a control.

### Immunohistochemistry staining

Human glioma tissue samples or solid tumors removed from sacrificed mice were fixed with 4% formaldehyde. Paraffin-embedded tumor tissues were sectioned to 5-μm thickness and mounted on positively charged microscope slides, and 1 mM EDTA (pH 8.0) was used for antigen retrieval. Endogenous peroxidase activity was quenched by incubating the slides in methanol containing 3% hydrogen peroxide, and they were then washed in PBS for 6 min. Next, the sections were incubated for 2 h at room temperature with normal goat serum and subsequently incubated at 4°C overnight with a primary antibody (Abcam: 1:250 ROCK-1). Then, the sections were rinsed with PBS and incubated with a horseradish peroxidase-linked goat anti-rabbit or anti-mouse antibody, followed by reaction with diaminobenzidine and counterstaining with Mayer's hematoxylin. HE staining was performed at the pathology department of Qilu Hospital of Shandong University for free.

### Orthotopically xenografted glioma mouse model

For investigation *in vivo*, intracranial brain tumor xenografts were obtained by stereotactically implanting transfected U87 cells (1×10^6^ cells per mice) into the brains of five-week-old male BALB/c nude mice (Chinese Academy of Sciences, Beijing). Cultured U87 cells were infected with a lentiviral vector LV3-hsa-miR-584-3p inhibitor, LV3-hsa-miR-584-3p mimic or LV3-negative control (Genepharma, Shanghai) according to the standard protocol. The mice were randomly divided into three groups (control, miR-584-3p mimic and miR-584-3p inhibitor), with five mice in each group. The animals were imaged by T2WI-MRI every 20 days. In accordance with ethical guidelines, the mice were sacrificed when the final MRI showed a sufficiently significant difference between the groups on day 60. After sacrifice, whole brains were removed and fixed with 4% paraformaldehyde, dehydrated, and embedded in paraffin. Serial sections of 5-μm thickness were cut and stained with H & E and then microscopically evaluated. The handling of mice and the experimental procedures were conducted in accordance with the experimental animal guidelines. The experiments conformed to the Animal Management Rules of the Chinese Ministry of Health (documentation 55, 2001), and the experimental protocol was approved by the Animal Care and Use Committee of Shandong University.

### Statistical analysis

All experiments were performed three times. Statistical analysis and experimental graphs were generated using SPSS 17.0 and GraphPad Prism software. Descriptive statistics, including the mean ± SD, Student's *t*-test, non-parametric Kruskal-Wallis test for multiple comparisons, Mann-Whitney test for comparisons of two groups, Kaplan-Meier plots, the log-rank test, one-way ANOVA and Spearman's correlation test, were used to analyze the significant differences, and a *p < 0.05, **p < 0.01, and ***p < 0.001 were considered statistically significant.

## SUPPLEMENTARY MATERIALS FIGURES


