# Supplementary material for: MicroRNA-584-3p, a novel tumor suppressor and prognostic marker, reduces the migration and invasion of human glioma cells by targeting hypoxia-induced ROCK1
Source: Oncotarget. 2015 Dec 23;7(4):4785–805. doi: 10.18632/oncotarget.6735 (PMC4826243; doi:10.18632/oncotarget.6735)
Supplement: Supplementary file 1 [file oncotarget-07-4785-s001.pdf]

# MicroRNA-584-3p, a novel tumor suppressor and prognostic marker, reduces the migration and invasion of human glioma cells by targeting hypoxia-induced ROCK1

## Supplementary Materials

miR-584-3p inhibitor: 5'-AGCCUGGUUGGCCUGGAACUGA-3';

miR inhibitor NC: 5'-CAGUACUUUUGUGUAGUACAA-3';

miR-584-3p mimics: 5'-UCAGUUCCAGGCCAACCAGGCU-3',

antisense:5'-CCUGGUUGGCCUGGAACUGAUU-3';

miR mimics NC: 5'-UUCUCCGAACGUGUCACGUTT-3',

antisense: 5'-ACGUGACACGUUCGGAGAATT-3';

miR-584-5p inhibitor: 5'-CUCAGUCCCAGGCAAACCAUAA-3';

miR-584-3p mimics: 5'-UUAUGGUUUGCCUGGGACUGAG-3',

antisense:5'-CAGUCCCAGGCAAACCAUAAUU-3'.

HIF-1 α Forward: 5'-ATCCATGTGACCATGAGGAAATG-3',

Reverse 5'-TCGGCTAGTTAGGGTACACTTC-3';

ROCK-1 Forward: 5'-AACATGCTGCTGGATAAATCTGG-3',

Reverse 5'-TGTATCACATCGTACCATGCCT-3';

GAPDH: Forward: 5'-TCCATGACAACTTTGGTATCG-3',

**Supplementary Figure S1: All primers used in this study.**

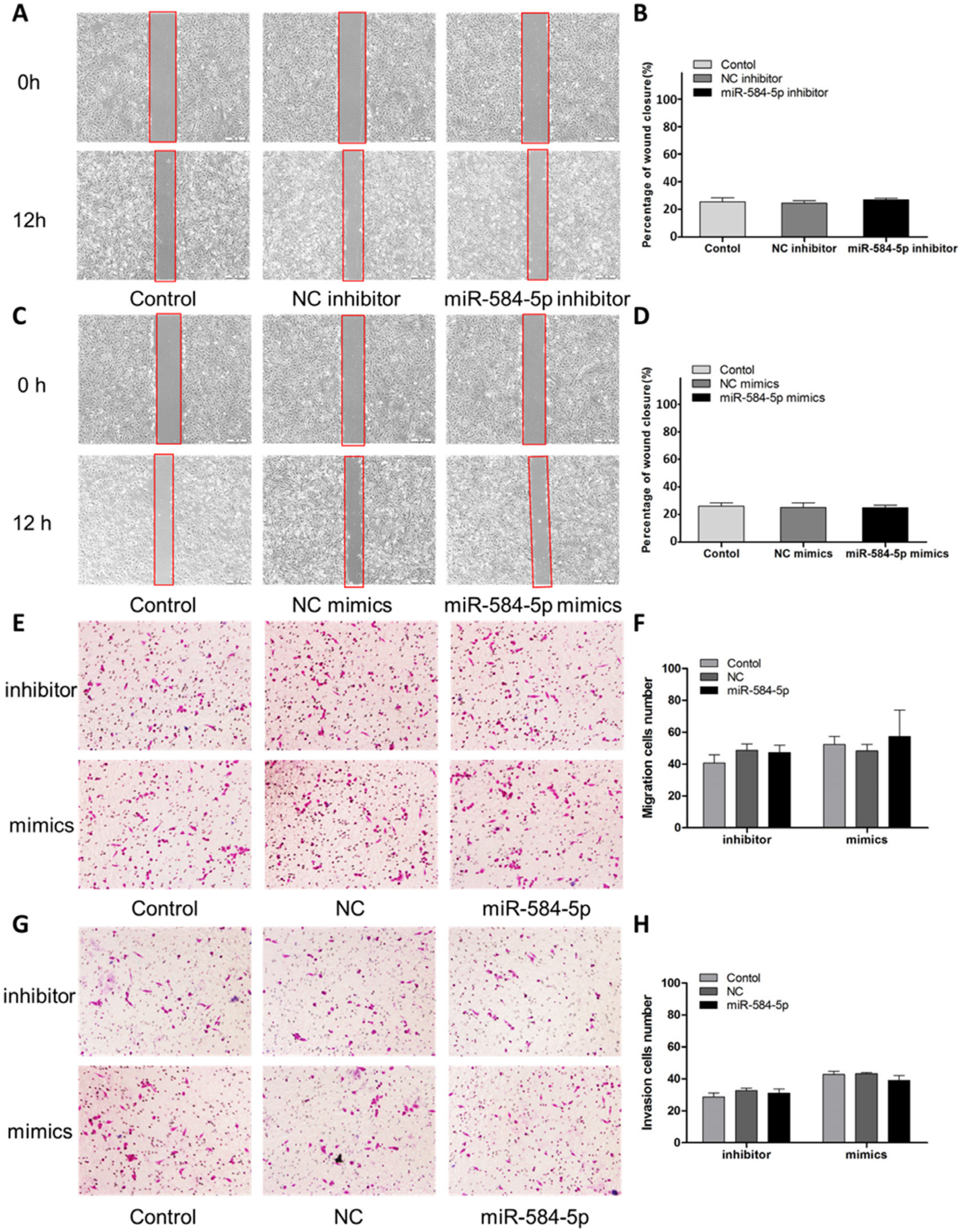


**Supplementary Figure S2: miR-584–5p had no effect on the migratory or invasive capacities of human glioma cells.** (**A** and **B**) Wound-healing assay of miR-584–5p inhibitor-transfected U251 cells. At 48 h after transfection, a wound was formed by scraping, and the wound was measured again after 12 h. (**C** and **D**) Wound-healing assay of miR-584–5p mimic-transfected U251 cells. (**E** and **F**) The effects of the miR-584–5p inhibitor and mimics on U251 cell migration were examined by Transwell migration assays. At 48 h after transfection, a cell suspension was added to the upper chamber of an uncoated Transwell membrane insert, and the lower chamber was filled with medium. The cells were cultured under normoxic conditions for 24 h. Then, migratory cells were stained, and the average number of cells was counted in triplicate. (**G** and **H**) The effects of the miR-584–5p inhibitor and mimics on U251 cell invasion were examined by Matrigel invasion assays. At 48 h after transfection, a cell suspension was added to the upper chamber of a 1:4 BD Matrigel-coated Transwell membrane insert, and the lower chamber was filled with medium. The cells were cultured under normoxic conditions for 24 h. Then, invasive cells were stained, and the average number of cells was counted in triplicate.
